# A Few Practical Hints on Sponge Tents

**Published:** 1871

**Authors:** Philip Adolphus

**Affiliations:** Chicago


A MONTHLY RECORD OF

*Medicine, Surgery and the Collateral Sciences.*

EDITED BY J. ADAMS ALLEN, M.D., LL.D.; AND WALTER HAY, M.D.

---

VOL. XXVIII.—OCT., NOV., DEC., 1871.—NO. 12.

---

### Original Communications.

ARTICLE I. — *A Few Practical Hints on Sponge Tents.* By PHILIP ADOLPHUS, M.D., Chicago.

(FIRST PAPER.)

It is well known that previously to the introduction of the uterine sound and the sponge tent by Simpson (1848), the interior of the uterus was a terra incognita to us. Diagnosis was faulty and treatment imperfect. Conjoined manipulation did indeed point to the enlargement of the uterus, but the nature of its contents could not be ascertained, nor readily removed. Patients then died of exhausting hemorrhage, the result of polypi and fibroids.

The use of the sound (or its modifications, the probes of Sims and Emmet) now permits us to ascertain the direction and the length of the uterine cavity, and the relative situation of any enlargement, whilst the expansion of the uterine neck and body, by the sponge or laminaria tent, allows the introduced finger to map out the nature of the foreign substance and its attachments, and thus admits not merely of their accurate diagnosis, but of such treatment as in skillful hands is now reasonably certain in its results.

Moreover, the sponge tent, simple or medicated, as will be shown, is highly prized, not merely as a means of diagnosis; since, by its means, granulations, hypertrophied follicles of the mucous membrane, small polypi, and fibroids, are destroyed; certain cases of mechanical dysmenorrhœa, and chronic congestions and hypertrophies of the womb, are cured; the neck of the uterus, for the purpose of removing the products of conception, is expanded; and, finally, it prepares the parts in the first stage of the induction of premature labor, for Barnes' dilators and other procedures.

It is needless to describe the mode in which sponge tents are made. From Simpson to Ellis, every magazine article and treatise on diseases of women contained a narrative of their manufacture; they are now an article of commerce, and can be found for sale in the shops.

The writer does not know whether tents, devoid of disinfecting qualities, are extensively used at present; they can, however, be found in the shops. Their disgusting odor, and the offensive putrilage emanating from them when soaked in the discharges of the uterus, render them disagreeable to physician and patient, as well as to the latter, by reason of possible pyæmia, dangerous. This has always been considered a drawback in their use. Practical men have regretted their indispensability whilst prizing the advantages gained by their use.

Therefore such tents should be discarded when others may be had, which contain a disinfectant, such as carbolic acid, permanganate of potassa, etc. Those made after the formula of Ellis, by having threads saturated with carbolic acid passed through the centre of the sponge, certainly are admirable; and if, after their insertion, a suppository of cotton wool, saturated with glycerine, is placed on the os uteri, as first recommended by Sims,* no fetor is perceptible either in the sponge or cotton wool when withdrawn; the putrilage generally emanating from the discharge of the uterus, and which is otherwise found in the vagina, is likewise absorbed by the cotton wool; and this, at most, has a musty smell. Occasionally, when a tent has not been sufficiently charged with carbolic acid, the odor is not entirely set aside, but it is so much modified by the glycerine that it cannot be distinguished by the patient, nor is it annoying to the practitioner.

* Clinical Notes on Uterine Surgery; T. Marion Sims, fol. 50.

The profession is greatly indebted to Robert Ellis, who, in 1867, introduced the carbolized sponge tent into practice,* for now the only objection to their use is removed.

Nos. 1, 2, 3, 4, 5, being respectively 1 inch, 1 1-4, 1 1-2, 1 3-4 and 2 inches,† are best adapted for the treatment of chronic uterine disease; for expanding the canal of the cervix, and for the removal of small polypi; but when the uterus is much enlarged by polypi or fibroids, Nos. 6 and 7 of the size of 2 1-4 to 2 1-2, and even larger sizes, are indicated. For large fibroids, it is best to use laminaria tents, in the manner to be described in a future paper.

The well-selected tent should be smooth, spindle-shaped and conical, so that it may adapt itself to the canal which it is to dilate; its calibre must also be suitable to the case for which it is intended, for a tent which is well adapted for one is unfit for another variety of cervix uteri. Neither must it be too long, for then it will impinge on the fundus uteri; in either case irritation and pain are likely to be the result of the introduction of a badly-fitted tent into the cervix by main force. It should, moreover, be perforated at its base for half an inch or an inch, for the purpose of its transfixion by the sponge-tent carrier; and, furthermore, the obnoxious string of the commercial sponge tent, which is found at its base, and by which it is intended to be removed from the uterine cavity, should be separated.

The physician should always have a number of tents of all sizes on hand, whenever he proceeds to their selection, for introduction. For the successful introduction and removal of sponge tents, the following instruments are requisite:

*a.* Sims' or Nott's speculum.
*b.* Sims' hook or Nott's vulsellum.
*c.* Emmet's probe.
*d.* Nott's dilator.
*e.* A sponge-tent carrier.
*f.* A uterine spring forceps.

The instruments under the captions *a*, *b*, *c*, *d*, have been described in previous papers, to which the writer refers. The sponge-tent carrier of Barnes, Hicks and Ellis, is a round mallea-

* Lancet, Sept. 7, 1867, page 295.

† The enumeration of H. Aveling. Obstetrical Transactions, Vol. 9, 1868, p. 267.

ble copper wire, eight inches in length, over which is pushed a tubular brass slide, exactly like that which is used with Emmet's applicator: this slide serves, when the wire is inserted into the extremity of a tent, to propel it into the cervix uteri.

The uterine spring forceps is used for the removal of the expanded tent from the os uteri. The tent is grasped by the forceps; a slide, propelled by a button on the side of the instrument, closes it. This self-retaining forceps holds the sponge until it is removed.

Lastly, but not least, a proper table or bed are essential to the success of this, as well as every other pelvic operation.

The writer will, therefore, give a few useful hints in regard to the table or bed most practical for this purpose, and will likewise add (as it is closely allied to the subject) the manner of making the ordinary pelvic examination, as this must precede the introduction of the sponge tent.

Whilst in office practice, Sims' chair, as described in his work, is the best that can be used, in private houses, a kitchen table, twenty-eight inches high, covered with a blanket or quilt, is preferable to the bed or couch, for it may be moved wherever the best light may be had, and it also permits the operator to sit comfortably whilst performing almost any operation. In many bed-rooms these advantages cannot be had, and even if light is present, the practitioner will have to kneel whilst operating, which is fatiguing.

Should a bed be used, it will be essential to place a leaf of a table or an ironing board under the matrass or blanket, to secure a non-yielding place for the pelvis, for non-success is almost certain where the pelvis buries itself in feathers or a resisting matrass.

Should a table be used, place a chair at its foot, the patient will mount it, sit on the edge of the table with her feet resting on the chair. She is then covered with a sheet, the practitioner supports her back whilst she lies down on the table; he lifts up her feet and places them on its edge, removes the chair, and sits immediately facing her covered pelvis. No assistant is necessary. Now let the practitioner take hold of the lower end of the sheet (which should always be of sufficient length to permit the patient to cover her face, should she desire to do so), state to his patient that her person is protected, and bid her to draw up her linen towards the upper part of her person. This will prevent exposure whilst the patient is obeying his injunction. (All kinds of fumbling by the practitioner about his patient's body linen is indelicate).

Having taught the patient to relax her abdomen, partly by gently expelling the air from her lungs, partly by drawing the abdominal parietes and contents towards the spinal column by a voluntary effort, the practitioner proceeds with the external examination of the abdomen; the examination per vaginam by the touch; the consentaneous examination with one finger in the vagina, the other hand externally; the introduction of Nott's speculum, and every succeeding step; all these are performed under cover. The examination of the abdomen and the pelvis by the touch are made whilst the physician is standing at the side of the patient; the introduction of the speculum whilst he is sitting.

Tents are introduced in the following manner: The patient having been placed either in the semi-prone position, or on the back, we ascertain the direction of the uterus by bi-palpation; introduce Sims' or Nott's speculum; steady the womb by Nott's vulsellum or Sims' hook; bend Emmet's probe, and introduce it into the uterus, ascertain therewith its length, sensibility and calibre. This is followed by the introduction into the os uteri of Nott's dilating forceps, which is gently expanded if necessary; not for the purpose of dilating the cervix, but in order to ascertain the *thickness* of the sponge tent to be introduced, just as the previous introduction of Emmet's probe has taught us the *length* of the tent to be selected.

This information thus gained will permit the proper selection of the tent, which must be freed of the string which is tied to its base. Bend the sponge-tent carrier to the exact curve of the cavity of the neck, push it into the orifice at the base of the tent (this will secure the tent to the carrier), moisten the tip of the tent with a solution of soap, and whilst steadying the womb with the left hand with the vulsellum or hook, insert the tent thus secured, with the right hand, gently into the neck, until all but a quarter of an inch has entered. Now drop the vulsellum, which, being self-retaining, remains in situ; then with the left hand, thus freed, push the brass coil or slide towards the neck of the uterus, while you withdraw the sponge-tent carrier. This movement results in lodging the sponge tent in the neck of the womb. All this must be accomplished without producing pain; nay, more, if the sponge tent causes inconvenience, pain or vomiting, it must be removed and a smaller one inserted, else mischief results. A suppository of cotton, saturated with glycerine, is then inserted into the vagina; a suppository of morphia and belladona is also introduced into the rectum; a napkin is secured by a T bandage, and the patient is ordered to bed.

A few cautions are here considered necessary, in order to avoid annoyance, insure safety, and ward off danger.

It is essential to ascertain the curve of the uterine cavity by means of the probe, else the tent will get entangled in one of the folds of the cervical mucous membrane, become saturated with fluids and rendered useless.

The uterus, in cases of flexions, should be straightened by means of probangs, armed with sponge, to render the introduction of the tent easy.

Let care be exercised that the tent be not pushed too far within the uterine cervix, else great difficulty and pain will be experienced in its removal.

It sometimes becomes necessary, owing to the non-dilatability of the internal os, which does not yield to the influence of the sponge tent, to use the knife, which is in these cases safer than metallic dilators.

The tent should be removed, if possible, the same day; Sims' rule of removing it in twelve hours is a good one, though it cannot in all instances be performed. Handled in the manner above described, they will neither produce inconvenience nor fetor, if left in situ for twenty-four hours.

Tents are introduced, and it is the constant practice of many physicians to insert them, whilst the patients are in their office. This is bad practice. Whilst it may often be done with impunity, cases will arise where misfortunes will compel the prudent practitioner to regret such a practice. Sims has described such cases, and every surgeon can add his experience in corroboration. They should never be used immediately before or after menstruation, else hæmatocele—a very frequent accident, when any operation, however trivial, is undertaken during that period—may occur. An effusion of blood into the pelvic organs may place our patient's life in jeopardy, and it will be only after many days confinement that this accident is recovered from. Cellulitis is another result of their use, if inflammation of the uterus, ovaries and tubes is present, or if the tents are too large, too frequently repeated, or too rudely inserted. Furthermore, sponge tents should not be used, when, in cases of flexions, adhesions have been formed by previous inflammations; as peritonitis may be induced. All chances of producing abortions should be avoided, by refraining from their use, until it is positively known that pregnancy does not exist.

The frequent accidents, occurring after the most harmless manipulations in pelvic surgery, in cases where the mishaps could be least expected to take place, should admonish us to use operative procedures only when absolutely necessary, and then with great deliberation. The writer has adopted the following rules, not insisted on in the text books, which will, on trial, be found useful: In chronic uterine disease he relies primarily on general medication; when, however, certain local and sympathetic troubles continue to manifest themselves, which by general consent are considered manifestations of pelvic disease, he uses local measures in conjunction. Should improvement take place, he continues to employ local treatment tentatively, or relinquishes it for the time being, still continuing general treatment; and does not resort to manipulations unless urgent symptoms necessitate their use.

Timely local measures, followed by constitutional treatment, will often break the vicious circle of ailments, which, without pause in their local treatment would but be perpetuated. *The surest index and guide in these cases is the improvement of the health of the patient;* excoriation of the neck, a retroverted or retroflexed uterus, accompanied by pain and the nameless neuralgic concomitants, require local treatment; the same state of affairs, without inconvenience, should not be disturbed. If we act otherwise, and produce hæmatocele, cellulitis, peritonitis, etc., and confine our patient, who, previously to our being called, had attended to her domestic affairs, to her bed for weeks and months, then our professional self-respect will be debased before the tribunal of conscience, and our pockets and reputation will experience a corresponding loss before that of the public.

The removal of the sponge tent is a delicate and important task. It is performed as follows:

Place the patient on her back or side, press the posterior commisure of the vulva back towards the anus, and with forceps remove the cotton wool suppository which will be found white, moist, of a musty odor (not foul or stinking). Introduce Sims' or Nott's speculum, lubricated, not with sweet oil or cold cream, but with fluid toilet soap, (which should always be kept in an open-mouthed phial, and with which the fingers during pelvic operations should be covered, whenever they are introduced into the vagina or rectum; for whilst it has no odor of its own, it, by its detergent qualities, purges the fingers of adhering impurities); the tent will be seen emerging from the expanded os uteri, dilated to four or five times the diameter it presented in its compressed state. Grasp it with the spring forceps, close the instrument, and leave it in situ whilst removing the speculum, by sliding it over the forceps. Now take the forceps in the right hand, introduce the index finger of the left hand into the vagina along its locked blades, let it sweep gently and firmly around the circumference of the cervix, whilst consentaneously an equally gentle rotatory movement is imparted to the forceps. This will free the sponge tent of its moisture, release the fibres of the sponge from the mucous membrane of the cervix in which it is imbedded, and leave the tent ready for removal in the grasp of the forceps.

The previous manipulations have been performed whilst sitting or kneeling in front of the pelvis; now stand on the right side of the patient, place the left hand on the abdomen, depress the uterus, whilst the index finger of the right hand is introduced into this organ; examine its interior carefully from cervix to fundus, perfect the diagnosis and perform the operation for which the uterus was dilated.

Should the cervix not be sufficiently expanded for the purposes of diagnosis and treatment, introduce another and larger tent, with the same precautions as above detailed.

In some cases the cervix does not permit the entrance of even the smallest sponge tent; here commence the expansion by a very small Bay of Fundy laminaria tent, dipped in warm water, which softens and lubricates it,* and on its removal, this may either be replaced by others of a similar kind, or a larger sponge tent.

Practically we have in this country no choice between the use of the sponge and laminaria tents; for whilst all sizes of excellent carbolized sponge tents may be procured with ease, the shops are but meagerly supplied with an imported article of the English sea tangle, which expands slowly and irregularly, and is withal to be had of a very limited range of lengths.

The Bay of Fundy tangle,† however, is an excellent means of

* Am. Journal of Obstetrics, J. C. Nott, Nov. 1870.

† For an excellent article on this subject see Nott on Laminaria Tents, above quoted.

expansion; but this again is supplied in limited quantities and lengths. Moreover, whilst it requires only one sponge tent, a faggot of two, four or six of laminaria tents is sometimes necessary for the same purpose, and as the price of either article is the same, the tangle tent will be used only in exceptional cases.

The use of the sponge tent as a diagnostic agent has occupied the attention of the writer thus far; the therapeutics of the sponge tent will receive his attention in a succeeding paper.

